# The influence of freeway curve characteristics on drivers’ speed perception accuracy

**DOI:** 10.1371/journal.pone.0267250

**Published:** 2022-05-04

**Authors:** Jinliang Xu, Haoru Li, Xiaodong Zhang, Fangchen Ma, Zhenhua Sun

**Affiliations:** 1 School of Highway, Chang’an University, Xi’an, Shaanxi, Peoples R China; 2 Chang’an University Journal Center, Xi’an, Shaanxi, Peoples R China; 3 Shaoxing Transportation Investment Group Co. LTD, Shaoxing, Zhejiang, Peoples R China; Southwest Jiaotong University, CHINA

## Abstract

Owing to the complicated geometric conditions and increasingly diversified driving environment, freeway curves have become a road section with frequent serious accidents. To ensure safe driving on curves, drivers must first perceive the movement condition and the vehicle’s position, and accurately evaluate the characteristics of the road to make the right speed choice. In this process, the perception of speed plays a crucial role. The present study aims to investigate the driver’s speed perception characteristics with respect to freeway curves to better understand the driver speed selection mechanism. We first construct six three-dimensional (3D) virtual highway models, which are consistent with the geometric lines and traffic engineering instruments of real freeways. A virtual simulation test is then conducted in a highly immersive environment. After the completion of the simulation experiment, we conduct a field verification experiment, test the actual driving speed and perceived speed of drivers in the same place as the simulation experiment, and verify the effectiveness of the simulation method. Finally, 25 3D curve models of four different types are constructed, and the influence of curve characteristics (e.g., curve radius, curve combination, tangent length between curves) on drivers’ speed perception accuracy are tested on the simulation platform. The results show that the driver’s perceived speed is lower than the real speed when driving on curves, and the tangent-to-spiral (TS) point is the section where the driver’s speed is most underestimated. Radius was the most important factor affecting driver’s speed perception, but the tangent length between curves also had a lesser influence. However, the curve combination had no effect on it. Our findings can help researchers and road designers understand the reasons for drivers’ speed choice, thus promoting drivers’ safety on freeway curves.

## Introduction

Owing to the complicated geometric conditions and increasingly diversified driving environment, it is well documented that freeway curves are the site of frequent serious accidents [1–4]. Council et al. evaluated the US National Highway Traffic Safety Association Fatality Analysis Reporting System (FARS) data, finding 29.5% of all fatal crashes to be speeding-related. Additionally, FARS data show that 54% of speeding-related rollover/overturn, jackknife, or fixed object crashes are on curves [5]. To ensure safe driving on curves, drivers must first perceive the movement condition and the vehicle’s position and accurately evaluate the characteristics of the road to make the right speed choice. In this process, the perception of speed plays a crucial role [6,7]. If drivers cannot properly perceive the speed of the car on a curve, they may be driving at an inappropriate speed, causing accidents.

As early as 1916, scholars discovered that drivers’ speed perception was usually inaccurate. At that time, scholars generally referred to the driver’s perception behavior as speed estimation or speed judgement [8,9]. Since then, scholars have conducted a number of studies on the phenomenon of inaccurate speed estimation by drivers. Generally, if the driver underestimates their speed then the speed will be higher than expected and vice versa [10]. Denton established a subjective perceived speed model of object movement, adopting the ratio method to obtain the perceived speed [11]. The model revealed the relationship between perceived speed and actual speed at a given speed and formulated the concept of speed adaptability for the first time. Schmidt et al. then proved that speed adaptability affects drivers’ speed perception: when drivers slow down after continuous high-speed driving, they significantly underestimate the speed [12]. This result was found to be consistent regardless of the diver’s experience and driving environment [13,14]. Conversely, Matthews and Michael [15] and Casey and Lund [16] found that drivers tend to overestimate their speed if they accelerate after maintaining a low driving speed.

The extant literature on influencing factors of speed perception mainly involves road alignment [17–19], road side environment [20], road structure [21,22], and driver characteristics [23]. Fildes conducted a multi-factor test, finding that drivers underestimate the speed of vehicles in curves, with experienced drivers having a higher speed perception accuracy, and radius having a significant impact on speed perception [17]. Recarte investigated the influence of speed, acceleration or deceleration behavior, and road alignment (tangent or curve) on passenger speed perception, showing that passengers’ speed perception deviation decreases with the increase of speed [18]. Stamatiadis found that road width, plane curve, and roadside trees strength had interactive effects on speed perception [19].

Nevertheless, there are still many gaps in the existing literature. First, the relationship between perceived and actual speed needs further study. Although many researchers have described certain patterns in which drivers underestimate or overestimate speed, they have only developed a general tendency, could not accurately describe the relationship between perceived and actual speed, and did not identify the variation characteristics of perceived speed in different road sections. Second, most studies were limited to whether a single factor has an impact on speed perception, with few studies on the impact degree.

In particular, previous studies on curves have confirmed that radius has a significant impact on drivers’ speed perception, but researchers only explored a small number of radius indicators, and the test were limited to a single curve. To identify drivers’ curve speed perception characteristics, many samples and tests are needed. At present, driving simulation is the main method used in relevant researches, and field tests are less used. When studying the speed behavior of drivers, the effectiveness of driving simulators can be guaranteed [24,25], but the effectiveness of speed perception studies has not been fully discussed. For example, Balligand found that the perceived speed in simulated driving was highly correlated with the perceived speed in real driving [26]; however, their conclusion was based on truck drivers rather than passenger car drivers and lacked comparisons with actual driving speeds.

Hence, this study first establishes a model consistent with the actual freeway curves on driving simulation platforms and verifies the effectiveness of the driving simulation method by comparing the data of driving simulation and field tests. Then, taking the radius, the curves’ combination (i.e., how two different curves are connected to each other), and the tangent length between curves as the research variables, we construct more comprehensive freeway curve models on the simulation platform. The actual driving speed and perceived speed data of drivers are obtained through driving simulation tests, and the speed perception characteristics of drivers in curves are explored. The novelty of this study is that it comprehensively considers the speed perception characteristics of various curve types and different curve feature points, thus having considerable significance for driving safety on freeway curves.

The remainder of this paper is organized as follows. The next section outlines the data and methodology. The Results section describes the analysis findings. The final section discusses the results and summarizes the conclusions.

## Methods

The experiment is divided into two overall parts: validity verification of the driving simulation method in speed perception research, and the analysis of speed perception characteristics based on the validity of the driving simulation.

### Validation test

To reveal the influence of curve characteristics on drivers’ speed perception characteristics, we relied primarily on driving simulation in this study. Hence, the validity of driving simulation must be analyzed. The curve radius, combination, and tangent length between curves are taken as variables for the selection of field test roads and establishing of simulation models.

#### Participants

We recruited drivers through an advertisement posted on the bulletin board of Chang’an University and a WeChat form. Through the way of inquiry, 18 drivers who had not used the driving simulator and were not familiar with the Xian-Xun freeway were screened out. Otherwise, All participants were in good health with normal vision (or corrected vision) and had no major traffic accidents. The participants were aged from 24 years to 52 years (M = 34.9) and comprised 13 men (72%) and 5 women (28%), which reflect the overall gender and age distribution of drivers in China. Meanwhile, their collective driving experience was between 2 to 25 years (M = 8.6).

The participants were informed of the general conditions of the test and signed an informed consent. The research was reviewed and approved by the Research Ethics Committee of Chang’an University, Shaanxi, China. The research content strictly follows the Declaration of Helsinki.

#### Equipment

**Fig 1:**
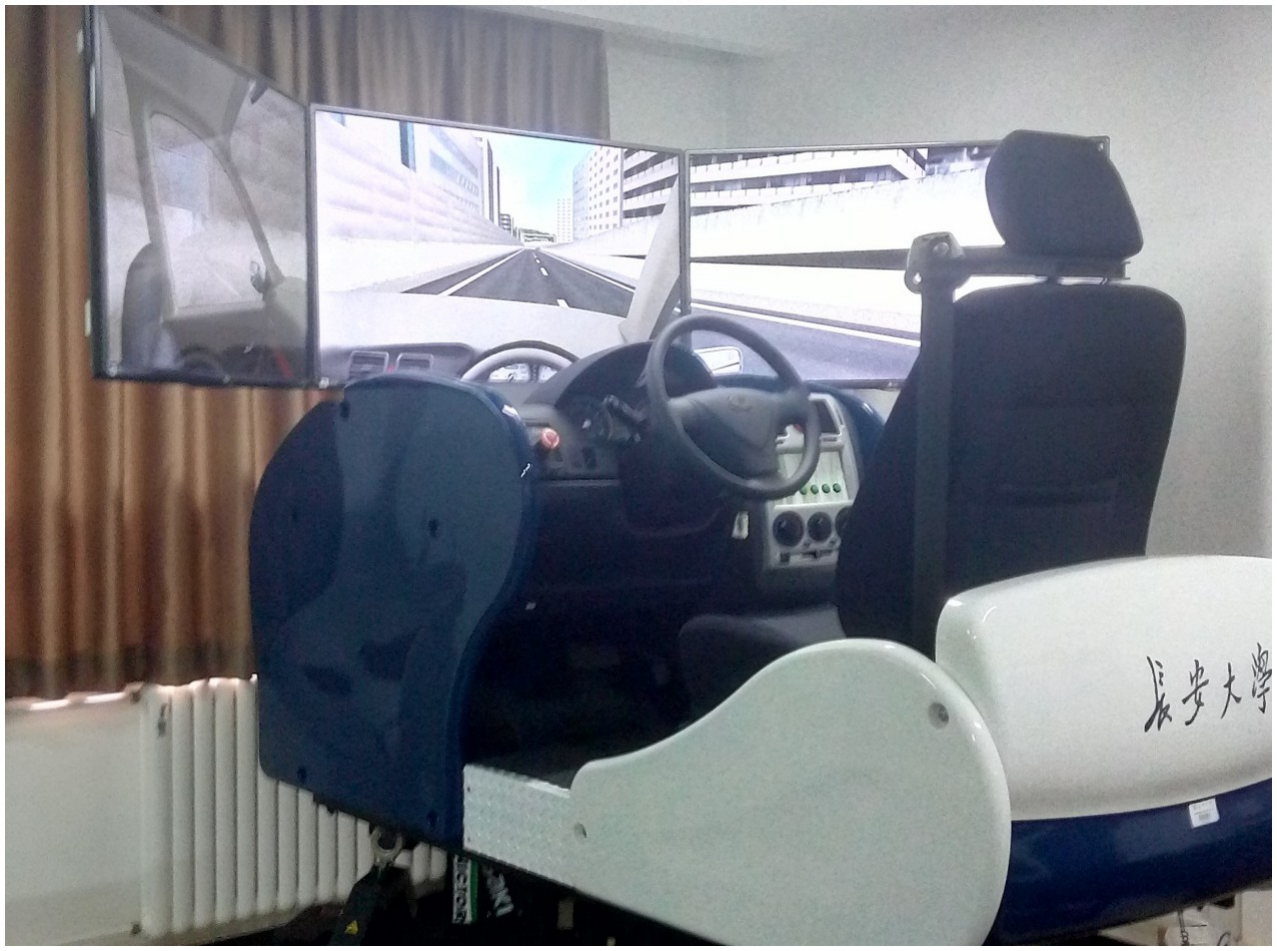
The 6-DOF motion platform.

**Fig 2:**
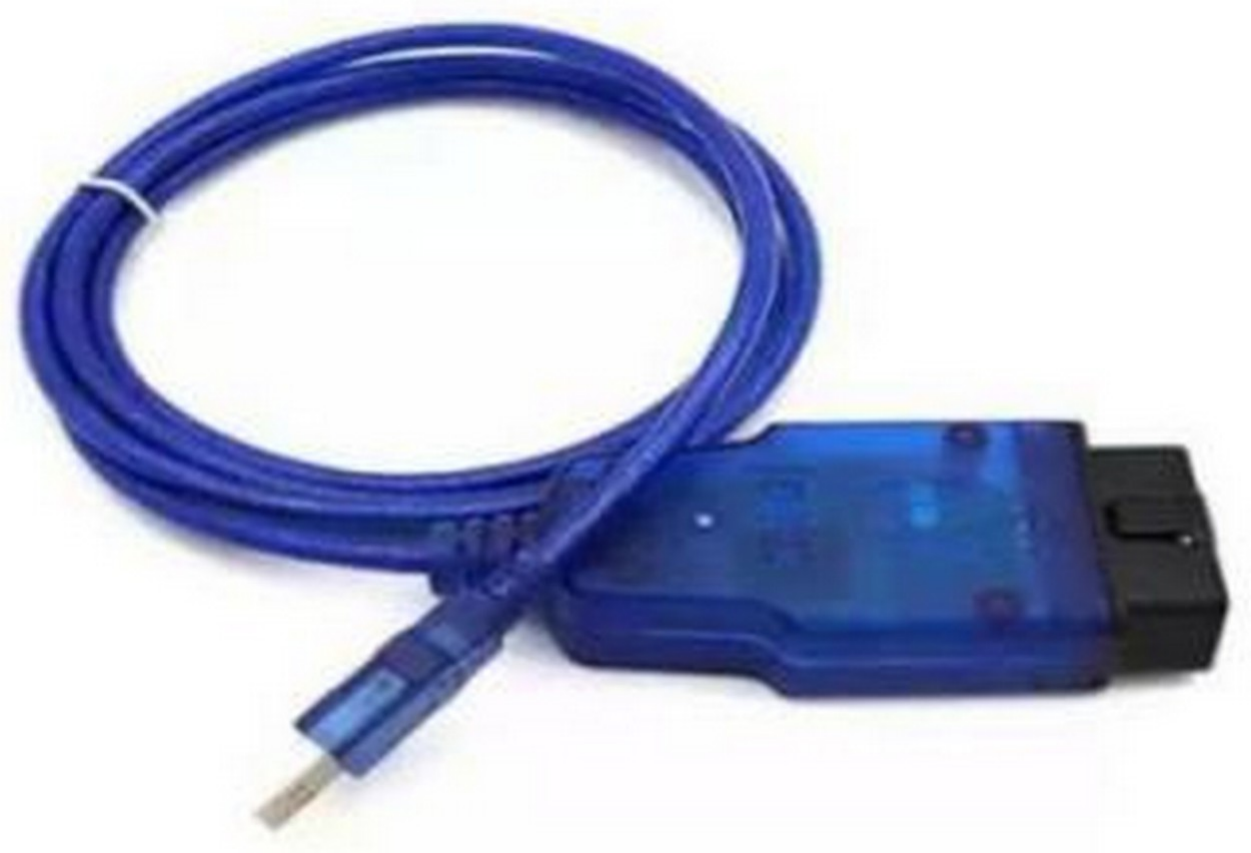
USBCAN-OBD converter.

The main test equipment of this study is a 6-Degree of Freedom (DOF) road driving simulation platform (shown in Fig 1) for driving simulation tests and an USBCAN-OBD converter (shown in Fig 2) for data transmission in the field test. The technical parameters are shown in Table 1.

**Table 1:** Summary of test equipment.

Facility	Technical parameter
6-DOF motion platform	The simulator system can provide a highly realistic virtual driving environment. The visual system provides a 130° horizontal and 40° vertical visual field in front. The sound system simulates the amplitude of the road and exhaust noises from other vehicles. The built-in motion system could realize the movement of 6-DOF in space, provides the drivers with a sense of acceleration, deceleration, steering, and sideslip movement. The data acquisition system could collect and output the driver’s operation behavior information in real time using a variety of sensors on the simulation platform, while its built-in ECO plug-in could obtain the speed data in real time.
USBCAN-OBD converter	This device was developed based on automobile Controller Area Network bus, which could obtain the vehicle speed data in real time and save it through the supporting software ECAN Tools in the computer.

The 6-DOF road driving simulation platform was developed by FORUM8 Corporation of Japan for the driving simulation test. The seats, steering wheel, dashboard, and shift lever used in the simulation driving system were all taken from the real vehicle.

The On-Board Diagnostics (OBD) refer to the self-diagnostic and reporting capacity of a vehicle. This adapter enables the vehicle network to be accessed by a computer. In service, it is similar to a computer modem or a gateway [27]. In the field test, the USBCAN-OBD converter was used as the speed acquisition equipment to convert the OBD data of the vehicle into USB data.

#### Test vehicle

The passenger car was chosen as the driving simulation model in this experiment for representability, and a Passat was selected as the field test car. Meanwhile, vehicle parameters were set in the UC-Win/Road software in accordance with the Passat applied in the field test.

#### Field road selection and simulation model establishment

**Fig 3:**
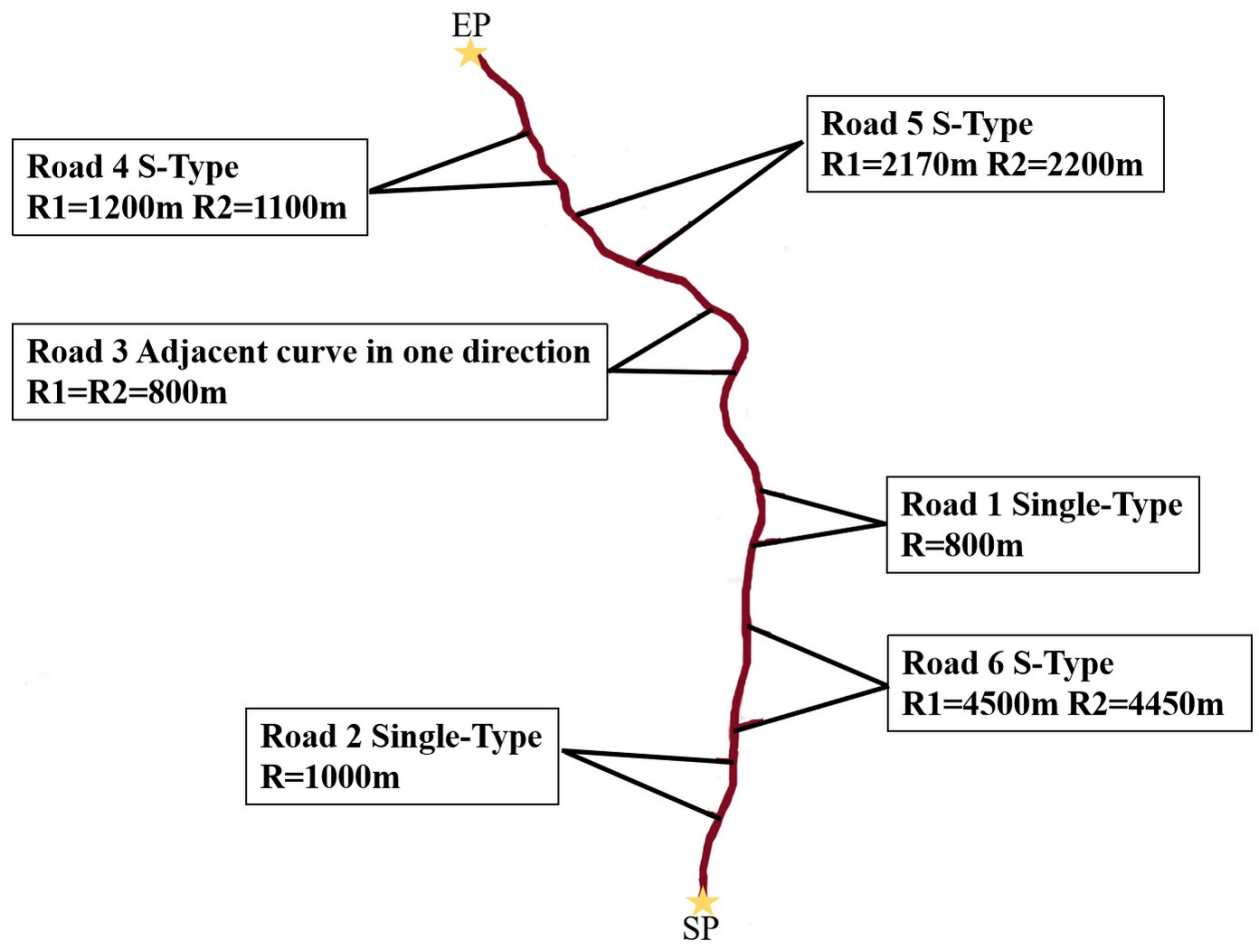
The 6 curves’ actual positions (SP means start point, was Xianyang North toll station, EP means end point, was Chunhua North toll station).

A field investigation was first carried out on Xian-Xun freeway. We selected six curves with monotonous roadside environment and gentle longitudinal slope. The spirals lengths were all between 200 m and 240 m. The specific design indicators were obtained through a design file, as shown in Table 2. The actual positions of the six curves are shown in Fig 3.

**Table 2:** Validation of test road parameters.

No.	Type	R(m)	L_f_ (m)	L_b_ (m)	Turing to Turing	i (%)
1	Single	800	1000	--	Right	-0.3
2	Single	1000	780	--		1.9
3	Adjacent curve in one direction	800–800	1470	370	Right to Right	<1
4	S-shape	1200–1100	600	--	Right to Left	1.54
5	S-shape	2170–2200	1835	--		0.3
6	S-shape	4500–4450	890	--		<2

R: The radius of the curve.

L_f_: The length of the tangent line before the curve.

L_b_: The length of the tangent line between curves.

i: The longitudinal slope of the curve.

**Fig 4:**
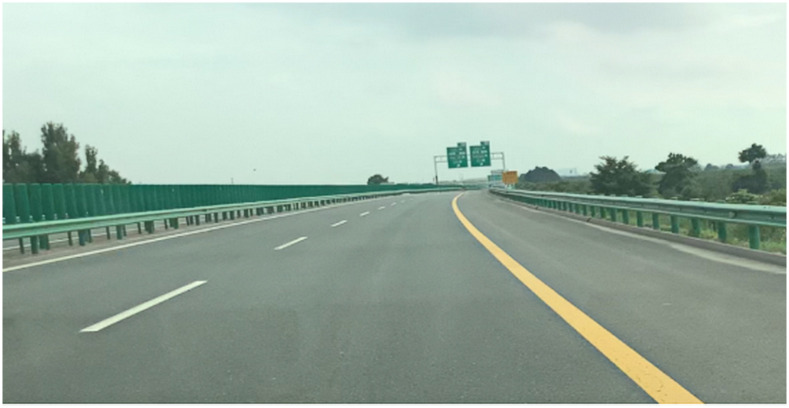
Schematic diagram of real road (S-shape curve).

**Fig 5:**
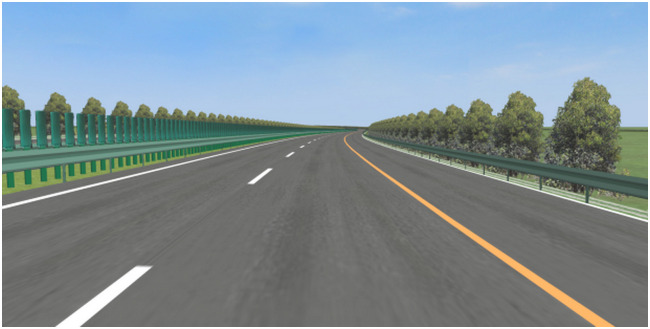
Schematic diagram of simulated road (S-shape curve).

The simulated road was established according to real road parameters, and the side environment of the simulation road is consistent with the actual road. In addition, a sunny weather and no other traffic flow were set to exclude the influence of the weather and traffic flow on the experiment. Figs 4 and 5 show comparison schematic diagrams of the real and simulated curves.

#### Perceived speed acquisition position and method

**Fig 6:**
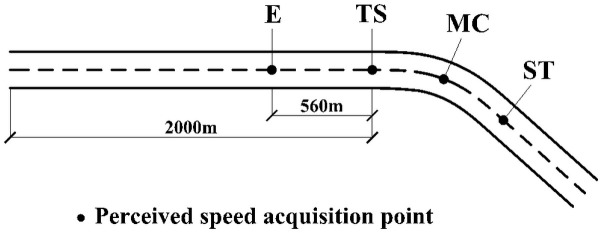
Single curve perceived speed acquisition point and horizontal alignment.

Islam and Seneviratne found that driving speed was different at different sections on a curve, including tangent-to-spiral (TS), midpoint of circle curve (MC), and spiral-to-tangent (ST) [28]. Hence, in this test, these characteristic points were selected as the acquisition points of perceived speed. Simultaneously, the geometric change when the curve appears in the driver’s visual field may affect the driver’s perceived speed. When the driving speed is 100 km/h, the driver’s gaze point focuses on the road about 560 m in front of the vehicle [29]. Therefore, the position where the curve enters the driver’s sight was defined as the exposure point E, and it was located 560 m before TS. The perceived speed acquisition points and horizontal alignment of the single curve are shown in Fig 6.

**Fig 7:**
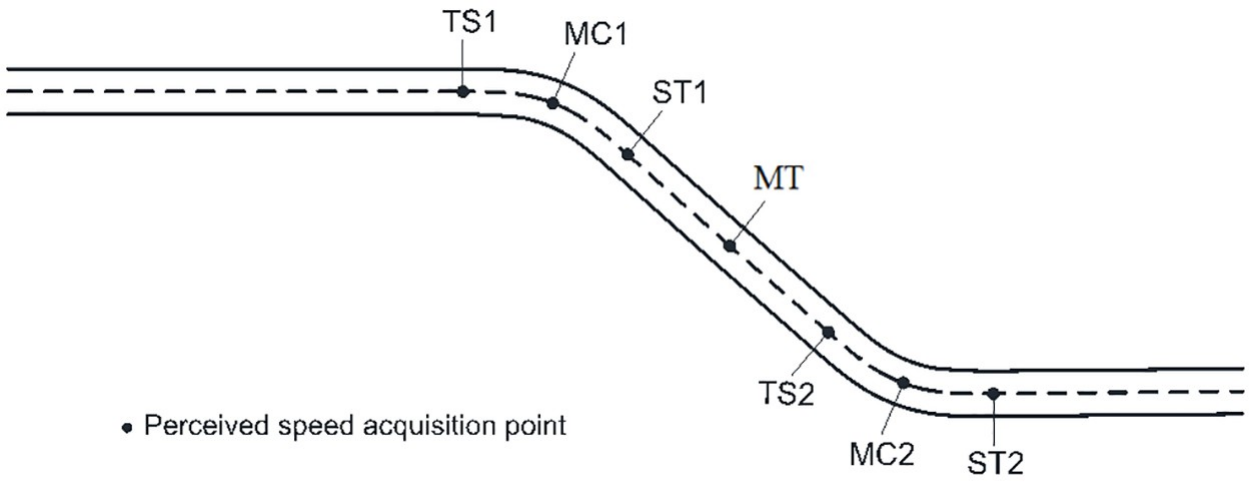
Reverse curve perceived speed acquisition point and horizontal alignment.

The perceived speed acquisition points of compound curves were similar to single curves, thus, the following elements were selected: TS1, MC1, ST1 (TS, MC, and ST points on the first curve, respectively), spiral-to-spiral (SS), midpoint of the tangent between curves (MT), TS2, MC2, and ST2 (TS, MC, and ST points on the second curve, respectively). The SS point only appeared in the s-shape curve, for the two curves of the s-shape curve were directly connected and there was no tangent between them. Taking a reverse curve for example, Fig 7 shows the perceived speed acquisition points.

In relevant studies on obtaining the real-time perceived speed of drivers, scholars primarily adopted two methods: "subjective equivalent speed method" and "self-report of drivers". The “subjective equivalent speed method” studied the relative relationship between the perceived speed and the actual speed of participants by playing two kinds of videos: standard scene and experimental scene. In this method, the subjects are only required to watch the video, which cannot reflect the real driving condition. The “self-report” method had been established in the field of criminology [30], which has been widely recognized in traffic safety research. However, it must be pointed out that the reliability and effectiveness of self-reporting methods are still of concern, as it is difficult to evaluate these characteristics without objective indicators of the same behavior [14]. In this study, we collected perceived speed data together with the actual driving speed, and checked its validity using comparative analysis. Hence, we adopted the “real-time self-report” method. Considering that the perceived speed obtained through self-reporting is highly subjective, it may have been possible for participants to believe that the more accurate the speed estimation, the better, which could lead to their attention being distracted by speed estimation rather than the real feeling of the road. Therefore, to avoid this, before the test we told the drivers: "Please drive according to your most authentic driving state. During the driving process, we will ask you some questions. Please answer as soon as possible as more accurate answers are not necessarily better.”

#### Procedure

**Fig 8:**
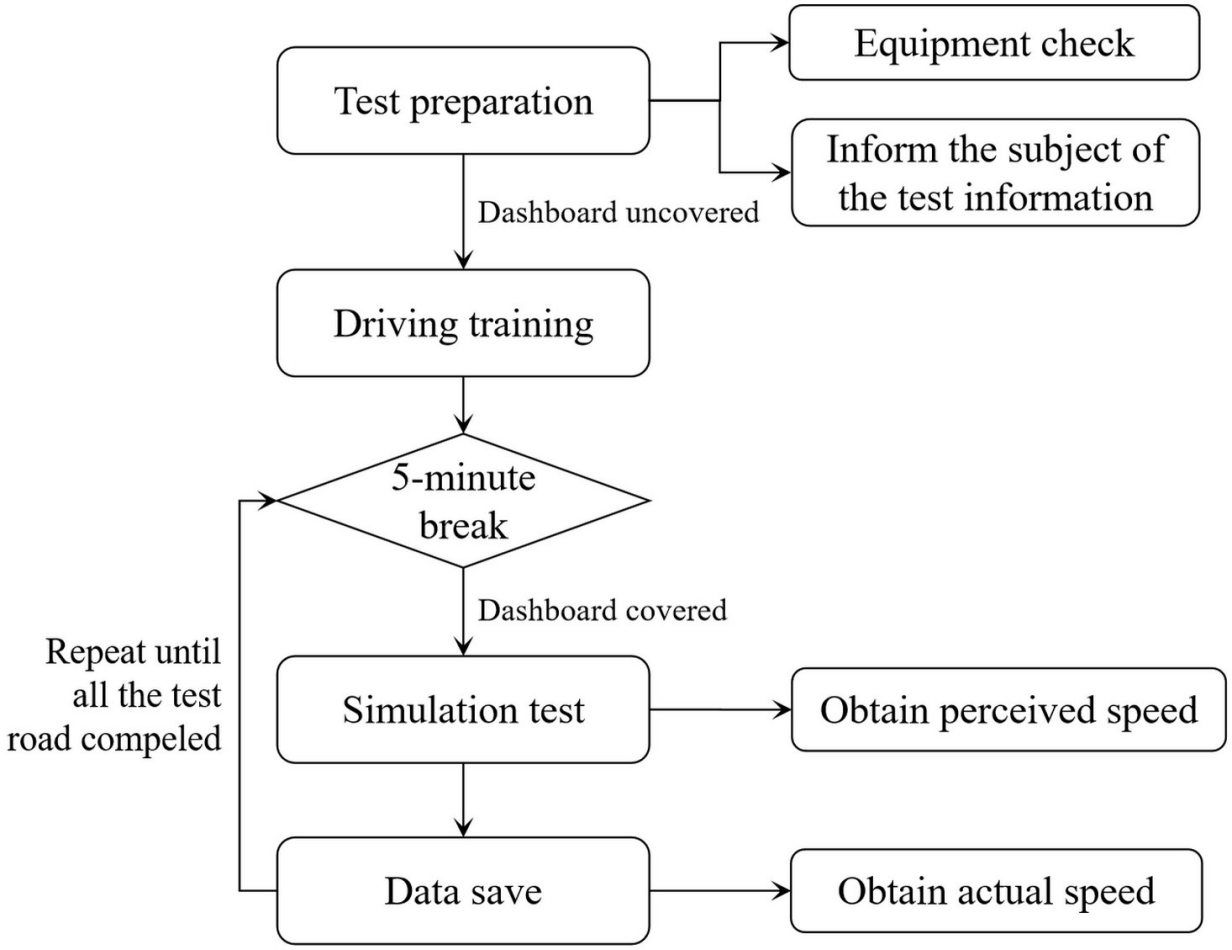
Validation test process (simulation test).

**Fig 9:**
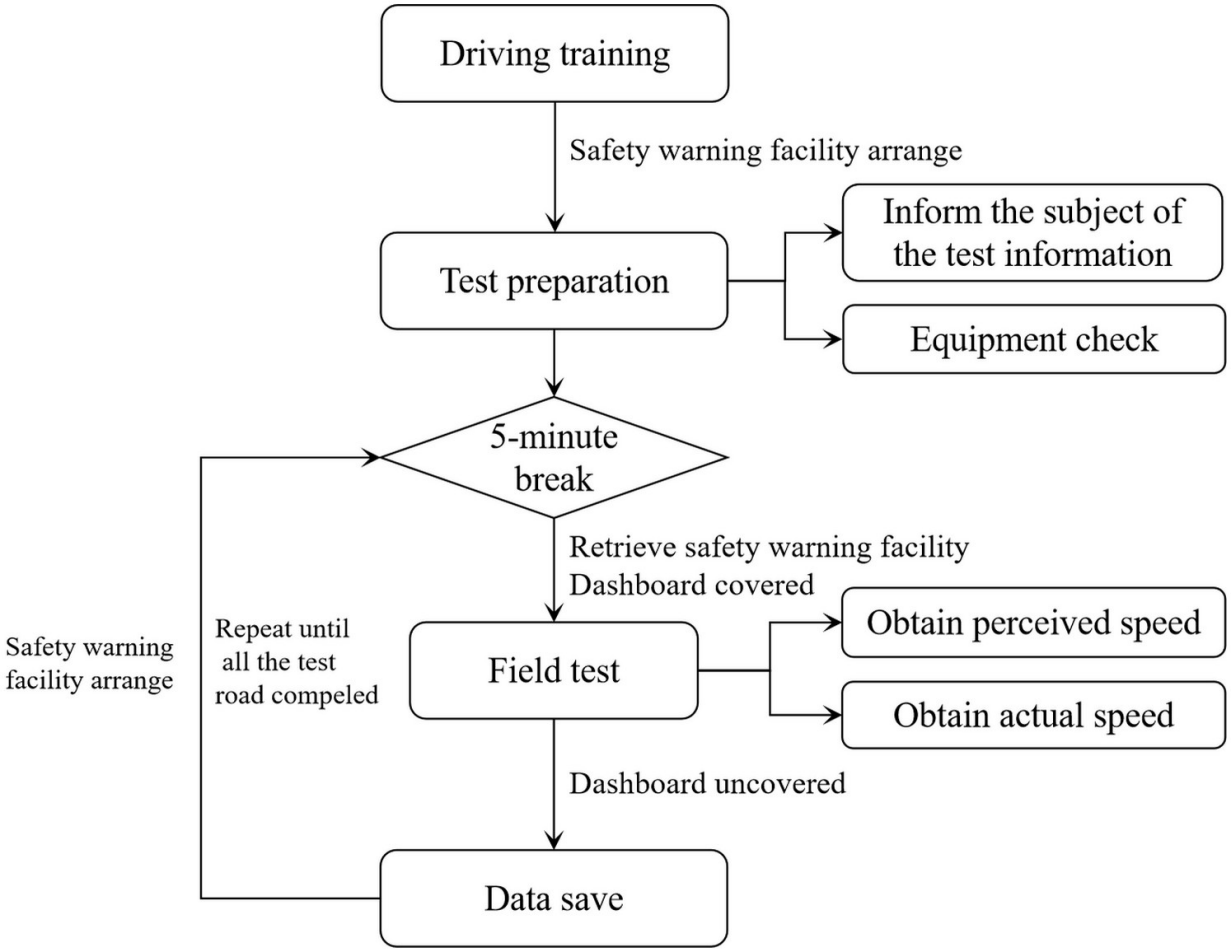
Simulation Validation test process (field test).

The validation test is divided into two parts: simulated driving test and field test. Both tests consist of three test stages: test preparation stage, driving adaptation stage, and road test driving stage. The specific test process is shown in Figs 8 and 9. The subjects were tested in turn and rested for 5 minutes after each road test to prevent fatigue.

For the simulation test, as the subjects needed to understand the operation of the driving simulator during the driving adaptation stage, a trial drive was conducted to adapt to the simulated driving environment and avoid blindly estimating the speed. The trial drive road was designed as a two-way, four-lane freeway with a 30 km length and 100 km/h design speed. There was no other traffic interference. The vehicle parameters, road longitudinal slope, cross-sectional elements, and other parameters were consistent with the test road. The drivers were told: “You can drive freely on the road and experience driving at different speeds through the dashboard.” After the trial drive, the drivers were asked if they felt dizzy or other discomfort to avoid driving simulator disease. The dashboard was also covered in the test road driving stage. The participants were told "Now, we will proceed to the formal test; you will not be able to know the actual speed through the dashboard. You can freely drive on the test roads but need to adhere to the traffic rules. The test road is a two-way four-lane freeway with 100 km/h speed limit. Please try to keep in the right carriageway near the hard shoulder. We will ask you some questions while you are driving and hope you answer briefly and explicitly." As drivers drove past the perceived speed acquisition positions, they were asked: "What’s your estimate for your speed now?" The test operator recorded the answer in the survey form. After the completion of each road test, the actual driving speed data in the simulator was stored, and the participants took a rest outside the test room for 5 min to eliminate the influence of the previous test on the driver’s speed perception and avoid visual fatigue caused by the display.

**Fig 10:**
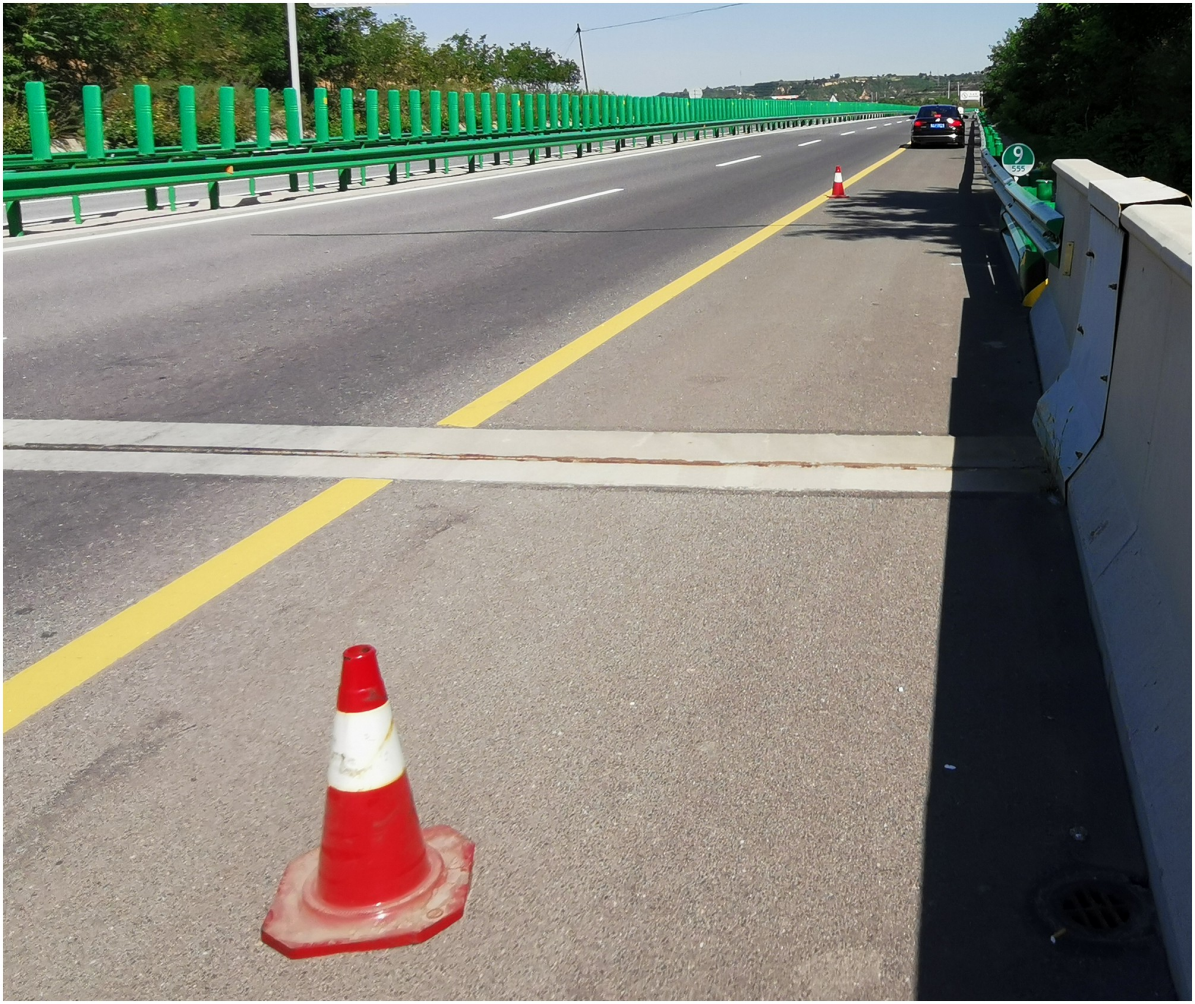
Real picture of field test.

The field test was conducted in a traffic-free environment on Xian-Xun freeway. The field test site was shown in Fig 10.

The participants drove the vehicle from Xianyang North toll station into The Xian-Xun freeway without covering the dashboard so that they could feel the speed while driving. Upon arriving at the start point of the first test curve road, the driver was prompted to stop. Then, the test operator wore a reflective vest and placed the warning triangles 100 and 150 m behind the vehicle for safety. The participants were told: “During the following driving, you will not be able to know the actual speed through the dashboard. If you drive too fast, we will remind you to slow down. You can freely drive on the road but need to adhere to the traffic rules. Please try to keep in the right carriageway near the hard shoulder. We will ask you some questions while you are driving and hope you answer briefly and explicitly." After the operator confirmed the speed acquisition software could work normally, the warning triangles were retrieved, the dashboard was covered, and the subject started the test. When the vehicle passed the perceived speed acquisition point, the operator asked the driver to perceive the speed and recorded it. Meanwhile, the actual speed was recorded. If overtaking or pressing occurred on the test road, which can affect the free driving of the vehicle, the test operator would indicate it in the record sheet. When driving off the test road, the operator stopped the speed recording software, saved the data, uncovered the dashboard, and let the driver drive to the beginning of the next test road where they stopped and rested for 5 min. When the vehicle stopped, the operator also placed the warning triangles at 100 m and 150 m behind the car to ensure the test safety. After a subject finished all 6 test road drives, the operator took the vehicle and the subject back to the starting point and the next subject performed the test.

#### Formal test

After confirming the validity of the driving simulation test method, the index parameters of radius, curve combination, and tangent length between curves were expanded to build a more comprehensive highway multi-type curve model for the driving simulation test, as shown in Table 3. The spiral length of all curves was set as 220 m and the curve length was set as 800 m, to prevent the curve length and spiral from influencing the drivers’ speed perception.

**Table 3:** Formal test curve simulation models index table.

**No**.	**Type**	**R(m)**	**Lf (m)**	**Lb (m)**	**Turing to Turing**	**i (%)**
1	Single	400	2000	--	Right	<2
2		500	2000	--		
3		650	2000	--		
4		800	2000	--		
5		1000	2000	--		
**No**.	**Type**	**R(m)**	**Lf (m)**	**Lb (m)**	**Turing to Turing**	**i (%)**
6	Adjacent curve in one direction	650–650	2000	400	Right to Right	
7		650–650	2000	800		
8		650–1000	2000	400		
9		650–1000	2000	800		
10		1000–650	2000	400		
11		1000–650	2000	800		
12	Reverse curve	650–650	2000	100	Right to Left	
13		650–650	2000	400		
14		650–1000	2000	100		
15		650–1000	2000	400		
16		1000–650	2000	100		
17		1000–650	2000	400		
18	S-shape	650–650	2000	--	Right to Left	
19		1000–650	2000	--		
20		650–1000	2000	--		
21		2000–650	2000	--		
22		650–2000	2000	--		
23		1000–1000	2000	--		
24		2000–2000	2000	--		
25		4500–4500	2000	--		

Only the driving simulation method was used in the formal test. The subjects, vehicle settings, test equipment, perceived speed acquisition method, and test procedures were consistent with the verification test.

### Data processing

The data of perceived speed and actual driving speed of 18 subjects were collected. From the analysis of the research status, the driver’s perceived speed was correlated with the actual driving speed. To analyze the variation characteristics of drivers’ perceived speed relative to the actual speed, the speed perception deviation (D) was calculated (for other curve types, D value was mainly used as the indicator to analyze the drivers’ speed perception characteristics) as

$$\text{D}=\frac{v_{p}-v_{t}}{v_{t}}\times 100\%$$ (1)

where *v*_*p*_ represents the mean value of drivers’ perceived speed, and *v*_*t*_ is the mean value of actual driving speed.

When D<0, drivers underestimates their own speed, whereas D>0 indicates that drivers overestimates their own speed.

## Results

The results include two parts: validation of driving simulation and analysis of drivers’ speed perception characteristics in different curve types.

### Driving simulation validation

**Fig 11:**
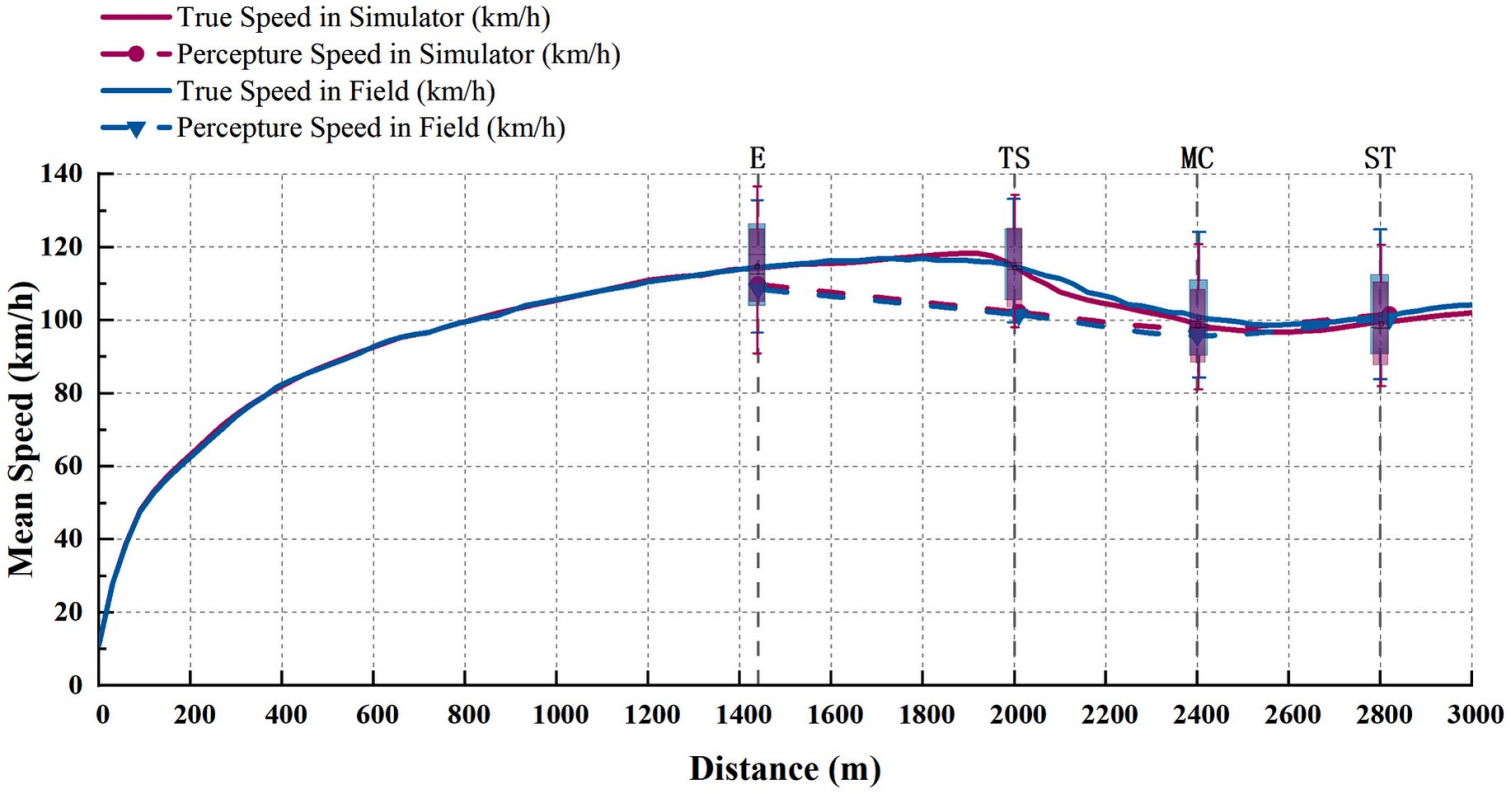
Comparison diagram of field and simulated mean speed data (The four longitudinal reference lines correspond to the perceived speed acquisition points: E, TS, MC, and ST).

Relative validity means that the driver’s driving behavior in the driving simulator is similar to the real road environment. Therefore, we compared and analyzed the actual and perceived speed values of vehicles on the road in the field test with those in the simulated test. Taking the single curve with a radius of 800 m as an example, Fig 11 shows a comparison of the continuous mean speed between the driving simulation and the field tests.

The results showed that the drivers’ speed behavior in the actual road has the same trend as that in the driving simulation environment. Additionally, the perceived speed at the characteristic points of the curve was also consistent in the two tests.

Considering the sample size of the test (only 18 drivers), a non-parametric test was used to verify the absolute validity. In this analysis, the Mann–Whitney (M–W) and Kolmogorov–Smirnov (K–S) tests were employed to verify whether the population mean of two samples was equal and the distribution shape was consistent. The specific analysis results are shown in Table 4.

**Table 4:** Mean speed comparison statistical test results.

Curve Type	Radius(m)	Point	Actual Speed				Perceived Speed			
			M-W Test		K-S Test		M-W Test		K-S Test	
			Sig.	Result	Sig.	Result	Sig.	Result	Sig.	Result
Single curve	800	E	0.815	Accept	0.766	Accept	0.673	Accept	0.964	Accept
		TS	0.696	Accept	0.814	Accept	0.481	Accept	0.491	Accept
		MC	0.462	Accept	0.766	Accept	0.339	Accept	0.491	Accept
		ST	0.864	Accept	0.766	Accept	0.389	Accept	0.964	Accept
	1000	E	0.628	Accept	0.27	Accept	0.839	Accept	0.971	Accept
		TS	0.963	Accept	0.766	Accept	0.963	Accept	0.964	Accept
		MC	0.171	Accept	0.131	Accept	0.265	Accept	0.131	Accept
		ST	0.104	Accept	0.131	Accept	0.239	Accept	0.131	Accept
Reverse curve	800–800	TS1	0.111	Accept	0.491	Accept	0.118	Accept	0.491	Accept
		MC1	0.481	Accept	0.766	Accept	0.444	Accept	0.057	Accept
		ST1	0.134	Accept	0.27	Accept	0.323	Accept	0.766	Accept
		TS2	0.839	Accept	0.964	Accept	0.424	Accept	0.964	Accept
		MC2	0.938	Accept	0.964	Accept	0.963	Accept	0.964	Accept
		ST2	0.181	Accept	0.27	Accept	0.462	Accept	0.971	Accept
S-shape curve	1200–1100	TS	0.323	Accept	0.491	Accept	0.696	Accept	1	Accept
		MC	0.252	Accept	0.766	Accept	0.293	Accept	0.766	Accept
		SS	0.143	Accept	0.491	Accept	0.104	Accept	0.491	Accept
		MC	0.743	Accept	0.27	Accept	0.181	Accept	0.491	Accept
		ST	0.339	Accept	0.131	Accept	0.232	Accept	0.964	Accept
	2170–2200	TS	0.791	Accept	0.27	Accept	0.424	Accept	0.814	Accept
		MC	0.864	Accept	0.964	Accept	0.563	Accept	0.964	Accept
		SS	0.265	Accept	0.766	Accept	0.323	Accept	0.491	Accept
		MC	0.443	Accept	0.491	Accept	0.888	Accept	0.964	Accept
		ST	0.308	Accept	0.766	Accept	0.462	Accept	0.491	Accept
	4500–4450	TC	0.424	Accept	0.964	Accept	0.104	Accept	0.131	Accept
		MC	0.743	Accept	0.766	Accept	0.239	Accept	0.27	Accept
		CC	0.542	Accept	0.814	Accept	0.279	Accept	0.27	Accept
		MC	0.542	Accept	0.491	Accept	0.913	Accept	1	Accept
		CT	0.462	Accept	0.766	Accept	0.743	Accept	1	Accept

The results showed that the absolute validity of the M–W test at 29 speed measurement points was demonstrated. The statistical analysis proved that the test results of driving simulation were highly consistent with the real scene in the curve. Hence, it may be concluded that it is reliable to use simulation tests to study the speed selection behavior and speed perception of drivers.

### Drivers’ speed perception characteristics analysis

#### Single curve

**Fig 12:**
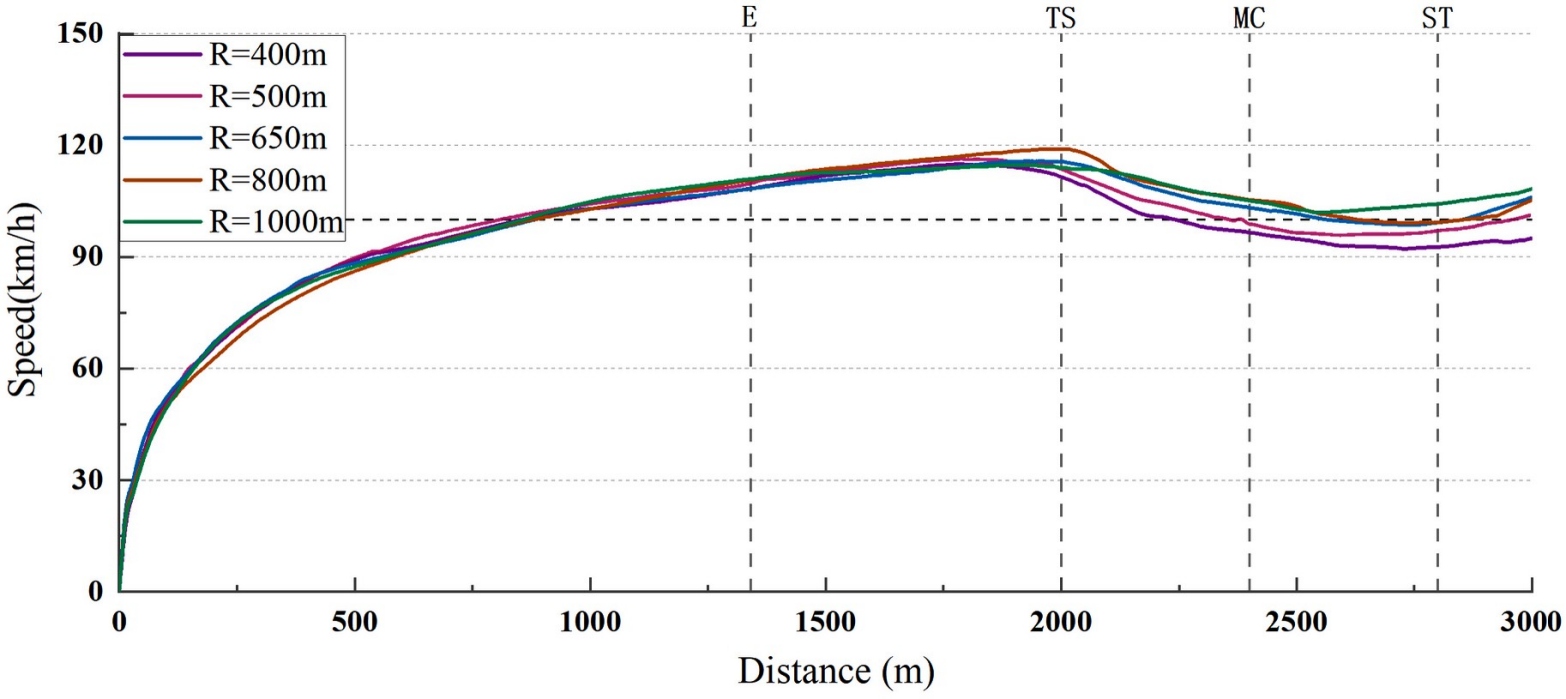
Drivers’ actual speed on single curves (mean value) (The horizontal dotted reference line indicates that the speed limit is 100 km/h; the four longitudinal reference lines correspond to the perceived speed acquisition points: E, TS, MC, and ST).

The actual speed and perceived speed of a total of 450 characteristic points of 18 subjects were obtained on the single curve. The drivers’ actual speed on single-type curves was drawn in Fig 12.

Based on the position of specific points on each curve, the speed change was analyzed. We found that the driver showed continuous acceleration behavior on the straight section where the driver had wide vision before the E point. After point E, the curve enters the driver’s field of vision, and the speed tends to stabilize gradually. Drivers generally began to decelerate at 100-200m before the TS point. The larger the radius, the closer this value was to the TS point. The lowest speed position was normally around the MC point, and then changed to an accelerated state at the second spiral. In general, drivers entered the curve at the same speed regardless of the radius, but with an increase in radius the drivers proceeded to slow down less. It can be seen that drivers generally traveled at a higher speed within the curve. On the curve with a speed limit of 100km/h, most drivers entered the curve at a speed exceeding the speed limit regardless of the radius, and at the same time, their speed decreased the most when they just entered the curve.

Pearson correlation analysis was conducted between drivers’ actual/perceived speed and radius to study the influence of radius on driver’s actual speed and perceived speed (conducted at the level of 0.05).

The results showed that the mean speed at point E (where the curve appears in the driver’s view) was moderately correlated with the radius (r = 0.558), while the perceived speed was strongly correlated with the radius (r = 0.719), but not significantly (Sig.>0.05). The mean speed and perceived speed of TS and MC were all significantly correlated with radius (r>0.900, Sig. <0.05). The mean speed of ST had a significant correlation with radius (r>0.900, Sig. <0.05), while the perceived speed had only a weak correlation with radius (r = 0.307). The correlation between the perceived speed and the radius of point E was surprising, although the correlation was not significant. We thought this may be due to the fact that most drivers drove at a high speed and could sense the curve ahead when they reached point E. Curvature changes may therefore have an effect on the speed perception of the driver.

**Fig 13:**
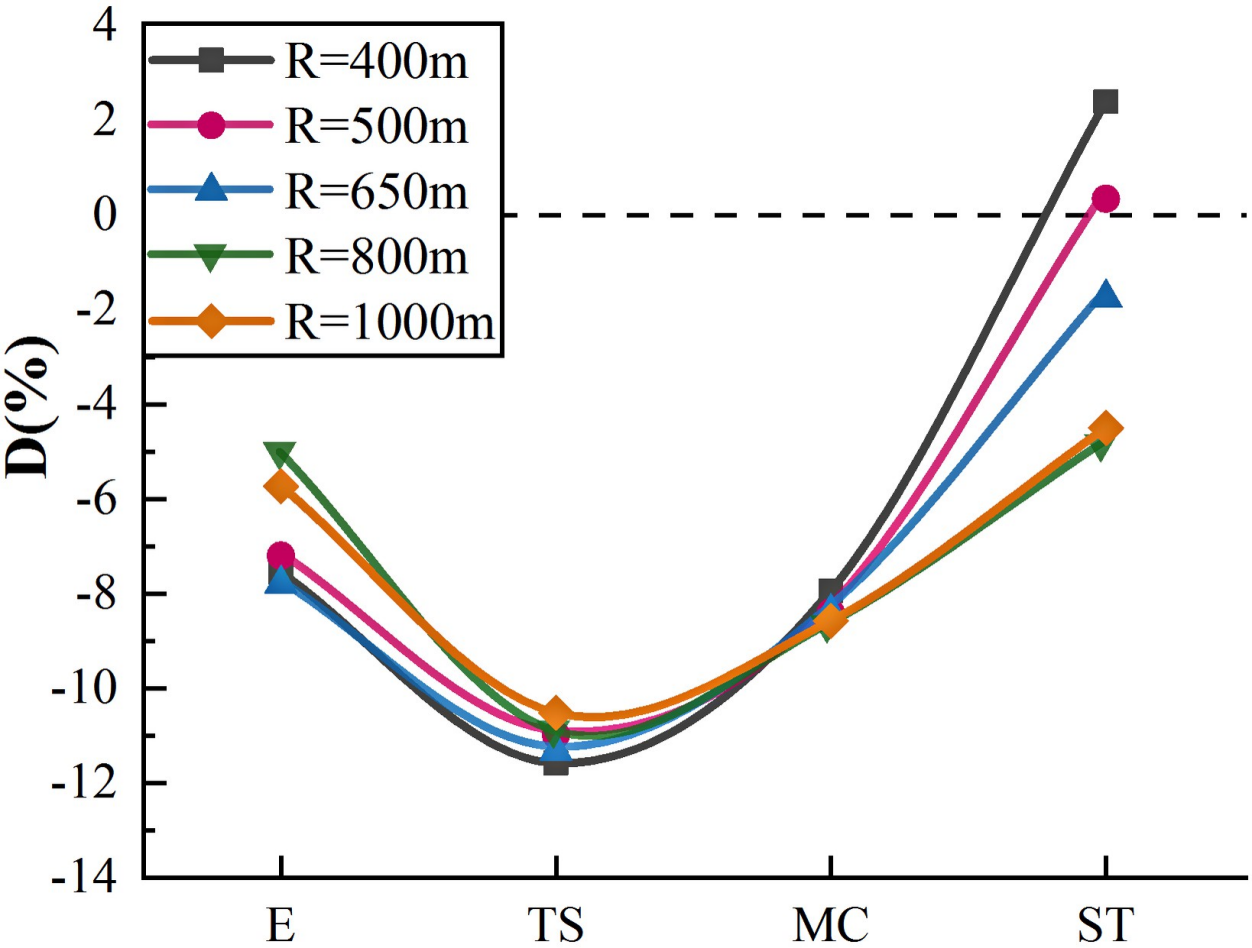
Perceived speed deviation diagram of single curve.

Calculated D for each point, as shown in Fig 13.

Fig 13 shows the U-shape relationship between the perceived speed deviation value and the perceived speed acquisition point. The D value was the lowest at the TS point, in which R = 400 m was the most underestimated (-11.57%), whereas R = 1000 m was the least underestimated (-10.51%). After the TS point, the speed degree underestimation was reduced. From TS to MC, drivers still underestimated the speed. Moreover, from MC to ST, the speed underestimation was significantly reduced and even slightly overestimated when the curve radius was small; the overestimation reached 2.40% when R = 400 m. Before the MC point, the D value did not show an obvious relationship with the radius; however, at the ST point, the larger the radius, the smaller the D value.

**Fig 14:**
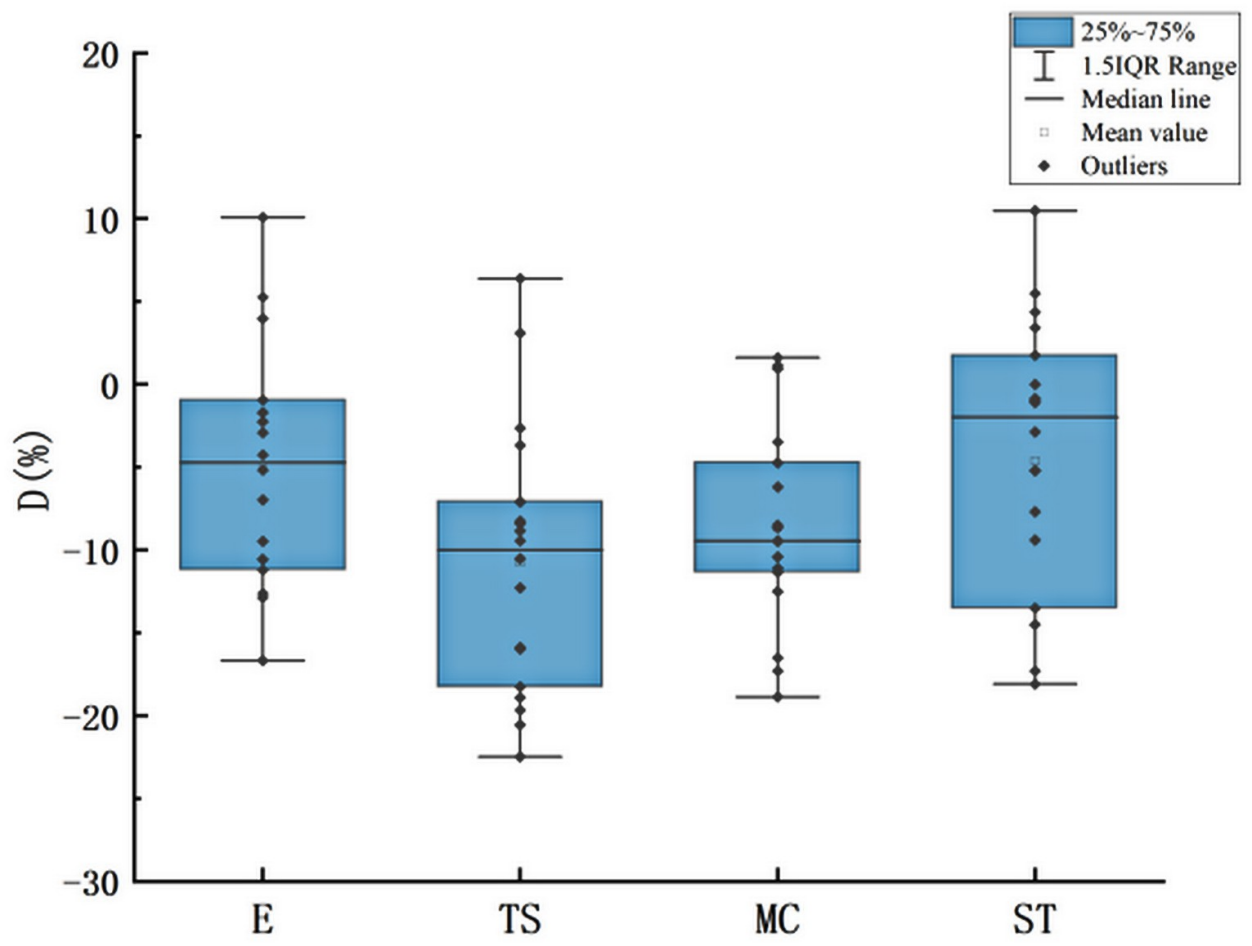
D-value distribution of 18 subjects (R = 800m).

To show the D-value distribution of the 18 subjects more clearly, a box diagram of D-value distribution was drawn for R = 800 m, as shown in Fig 14.

Fig 14 indicates that different drivers had different speed perceptions on curves. Most drivers underestimated the speed, whereas a few drivers overestimated the speed. Using the comparative analysis of the D values of the 18 subjects, we found that even drivers who overestimated their speed showed a trend of first decrease and then increase in the D values after entering the curve. That is, the influence tendency of curves on all drivers was consistent.

Correlation analysis was carried out on the perceived speed deviation of each point corresponding to different radii. The results showed that perceived speed deviations at points E and TS had a strong but not significant correlation with the radius (r>0.700). The D value showed a negative correlation with the radius (r = -0.836) at point MC, and a significant correlation with the radius (r = -0.936, Sig.<0.05) at point ST. From point E to point ST, the relation between the radius and D value changed from positive to negative correlation, indicating that curves have a significant effect on drivers’ perception of speed. The accuracy of the driver’s speed perception improved after the curve. When the radius is less than 650 m, the overall improvement of speed perception on the curve was strong, and the speed might even be overestimated when leaving the curve. When the radius was greater than 650 m, the perceived speed improvement rate on the curve was slightly slower, and the speed underestimation degree was still above -4% when drivers were leaving the curve (ST point).

During the test, the gender, age and driving experience of the subjects were collected. We wondered if the personal attributes of drivers had an impact on their speed perception. Therefore, the correlation between D value at each point and the personal attributes of drivers was analyzed. The results showed that when drivers driving in single curves with different radius, the correlation between their personal attributes and speed perception deviation was basically the same. The analysis results were shown in Table 5. (Take R = 400m single curve as an example.).

**Table 5:** Correlation between D value and the personal attributes (r).

Point	Gender	Age	Driving experience
E	0.445	0.136	0.429
TS	-0.046	0.458	0.676 (0.05)
MC	0.020	0.221	0.598 (0.05)
ST	-0.099	0.006	0.152

The results showed that there was no significant correlation between the gender and age of drivers’ personal attributes and the D value, while the driving experience and the D value showed significant positive correlation at TS and MC points. It indicated that drivers with longer driving experience would have a higher D value when driving in the curve. Drivers generally underestimate the speed (D<0) at these two points, therefore, it can be considered that more experienced drivers have more accurate perception of speed.

#### Adjacent curve in one direction

**Fig 15:**
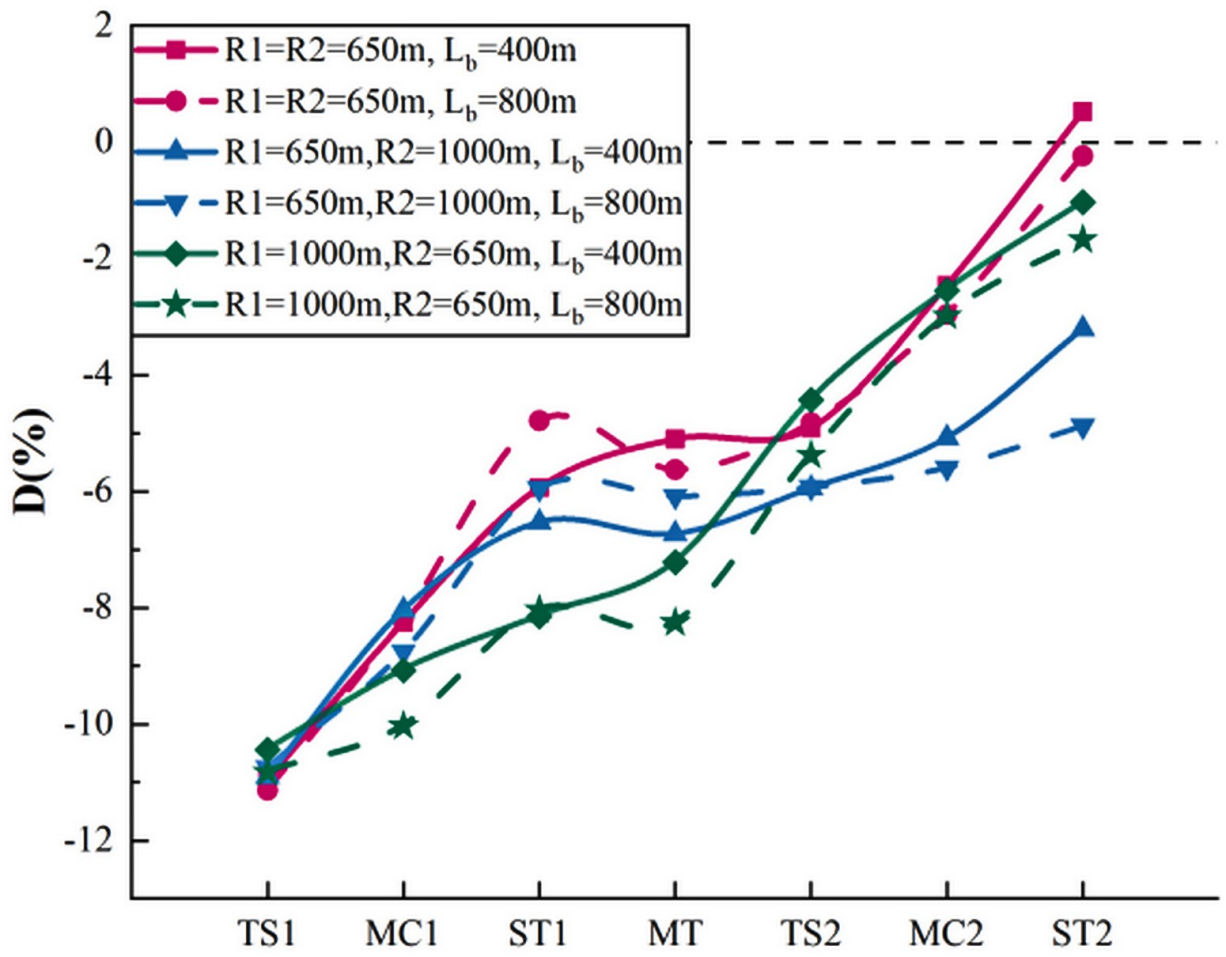
Perceived speed deviation diagram of adjacent curve in one direction.

The actual speed and perceived speed at a total of 450 characteristic points for 18 subjects were obtained on the adjacent curve in one direction. We recorded R1 as the radius of the first curve, R2 as the radius of the second curve, and L_b_ as the tangent length between the curves. Similar to single curves, the perceived speed deviation was calculated, and the deviation curve was drawn, as shown in Fig 15.

Fig 15 illustrates that the effect of the adjacent curve in one direction on the driver’s speed perception improvement was more significant when the tangent length was shorter. When the tangent length was long, the speed perception deviation at ST2 was large. The most improved speed perception effect occurred in the curve with a radius of 650 m and tangent length of 400 m, with the deviation at ST2 being 8.50% better than that at TS1. Meanwhile, the curves with radii of 650 and 1000 m and tangent length of 800 m had the weakest improvement effect (5.16%).

The Pearson correlation analysis was conducted between D value and radius ratio (R1/R2) and tangent length, with the results shown in Table 6.

**Table 6:** Correlation analysis of D of adjacent curves in one direction (r).

Point	Radius Ratio	R1	R2	L_b_
TS1	0.544	0.776	--	--
MC1	--	-0.857(0.05)	--	--
ST1	-0.71	-0.902(0.05)	--	0.225
MT	-0.929(0.01)	-0.836(0.05)	0.771	-0.146
TS2	-0.611	-0.836(0.05)	--	-0.146
MC2	0.681	--	-0.875(0.05)	-0.248
ST2	0.772	--	-0.981(0.01)	-0.194

The results showed that the D value at MC and ST points is significantly correlated with the curve radius (r>0.800, Sig.<0.05). The perceived speed deviation at all the points is not significantly correlated with the tangent length between curves. The D value at point MT is significantly negatively correlated with R1 and Radius Ratio. The D value at point TS2 is significantly negatively correlated with R1 but not with R2, indicating that when the driver traveled to the second curve, the speed perception was affected by the previous curve. Thus, the speed perception has transitivity between the adjacent curves in one direction; however, the transitivity loss threshold of tangent length needs further study.

#### Reverse curve

**Fig 16:**
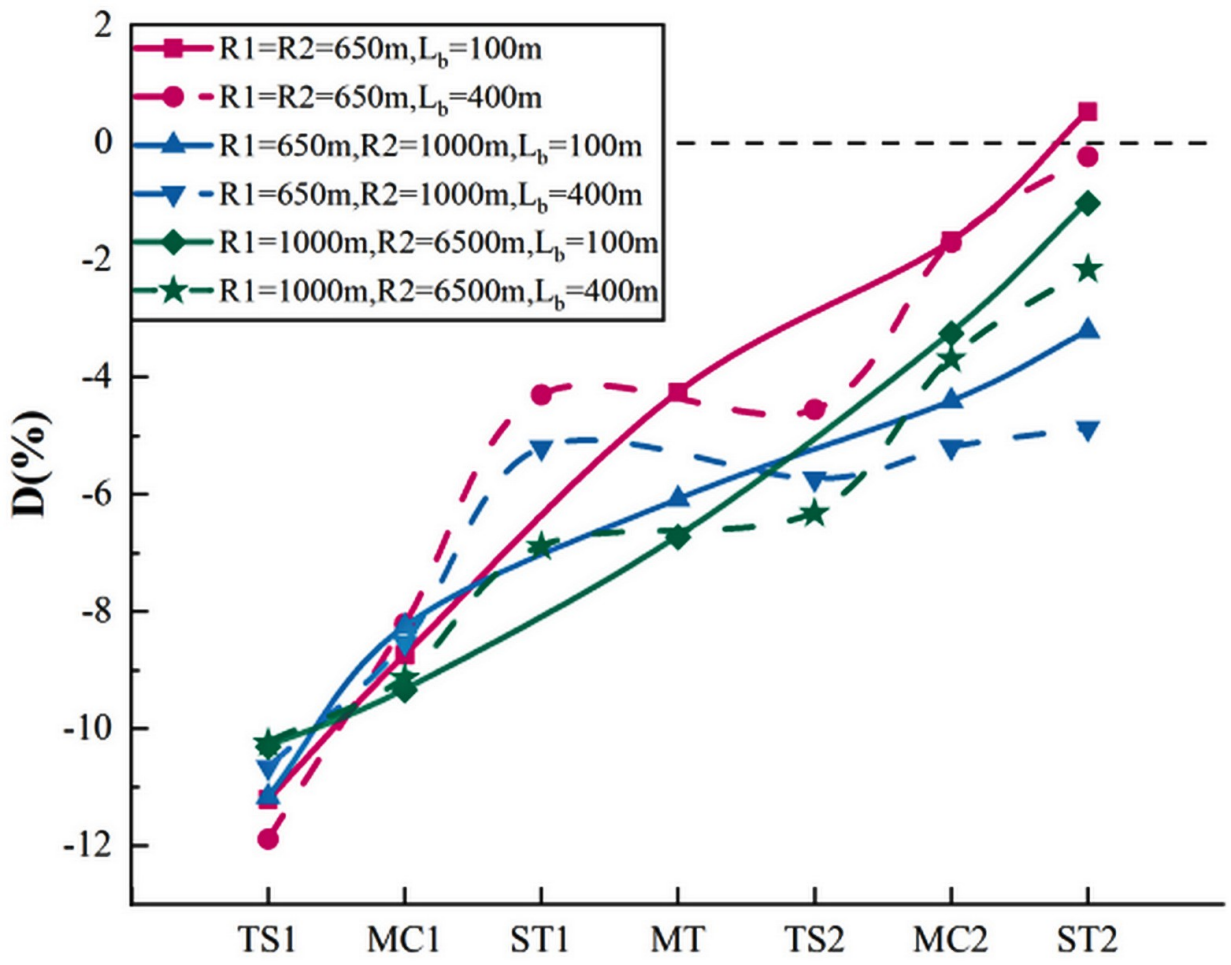
Perceived speed deviation diagram of reverse curve.

Similar to adjacent curves in one direction, we assumed that when the tangent length between the reverse curves was shorter, the curve had a more obvious trend with respect to improving the perception of speed. We calculated the perceived speed deviation and drew the deviation curve as shown in Fig 16.

Fig 16 shows that reverse curves also improved the driver’s speed perception accuracy, and that the effect was more significant when the tangent length was shorter. The deviation rule of speed perception was consistent with adjacent curves in one direction. The curve with a radius of 650 m and tangent length of 100 m had the best improvement effect. The deviation at ST2 was 11.74% better than that at TS1. The curve with the weakest improvement effect (5.78%) had radii of 650 and 1000 m and tangent length of 400 m.

Table 7 shows the results of Pearson correlation analysis conducted between D value and radius, D value and radius ratio, and D value and tangent length.

**Table 7:** Correlation analysis of D of reverse curves (r).

Point	Radius Ratio	R1	R2	L_b_
TS1	0.544	0.783	--	--
MC1	--	-0.898(0.05)	--	--
MT	-0.474	-0.771	--	0.105
MC2	0.303	--	-0.863(0.05)	-0.160
ST2	0.448	--	-0.855(0.05)	-0.325

The results showed that perceived speed deviation at MC1, MC2, and ST2 points is significantly and strongly correlated with the curve’s radius (r>0.800, Sig.<0.05), but not significantly correlated with the tangent length between the curves. Compared with adjacent curves in one direction, R2 has a slightly lower influence on the driver’s D value at MC2 and ST2 points, and the correlation between D value and R1 at the MT point becomes lower, indicating that the influence of radius on the driver’s speed perception was more obvious in the adjacent curves in one direction. Nevertheless, since the tangent length of the two types of measured curves was not controlled for successfully, the reliability of this specific conclusion needs further verification.

#### S-shape curve

**Fig 17:**
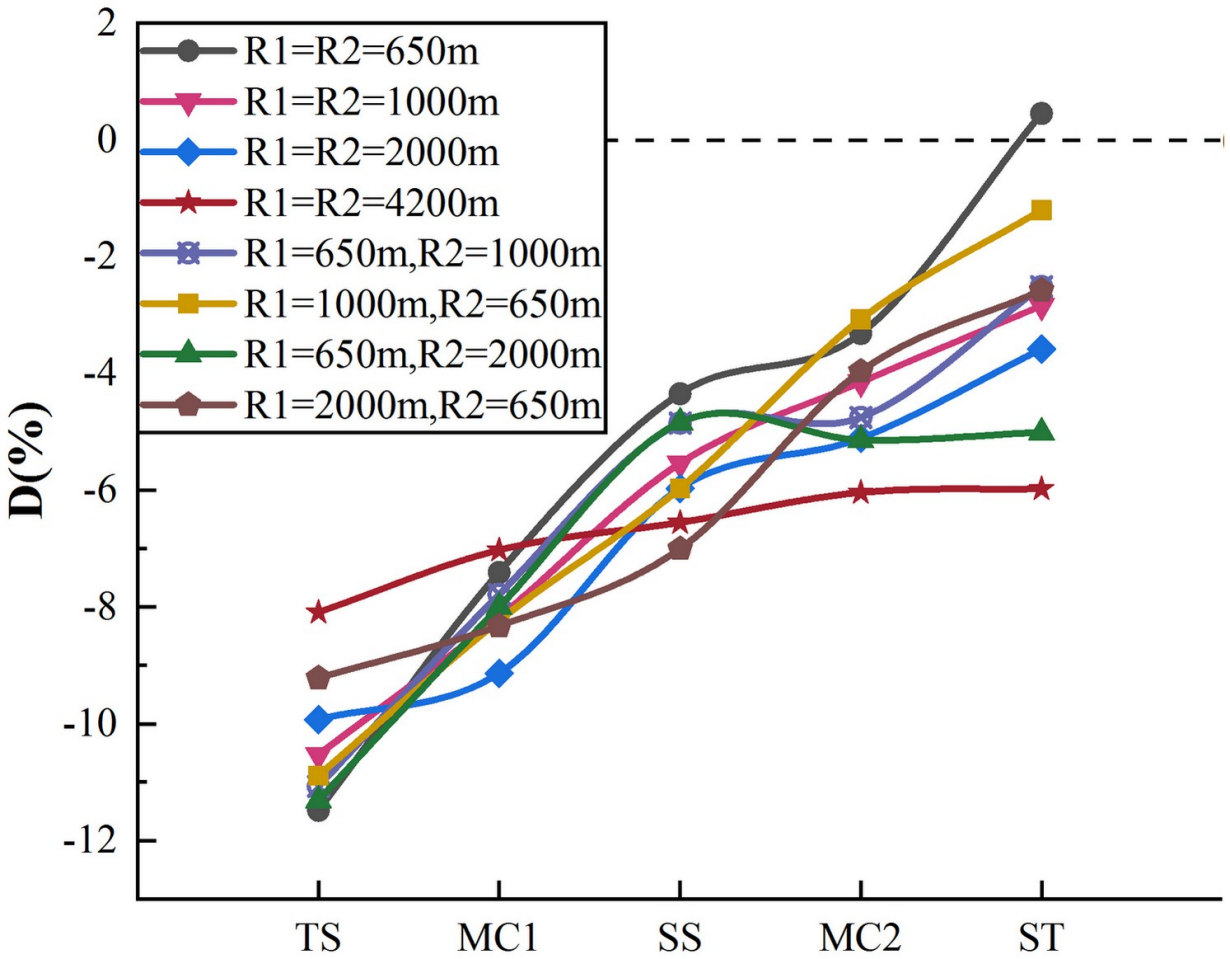
Perceived speed deviation diagram of s-shape curve.

The actual and perceived speed at a total of 720 characteristic points for the 18 subjects were obtained on the s-shape curve. Fig 17 shows the perceived speed deviation of the s-shape curve.

The improvement effect of the s-shaped curve on driver’s speed perception was similar to that of the reverse curve with shorter tangent length. The curve with 650 m radius showed the best improvement effect on speed perception deviation (the deviation at ST2 was 11.84% better than that at TS1), whereas the curve with 4200 m radius had the weakest effect (2.12%). Curves with a radius ratio greater than 1 were better than those with a ratio less than 1, while curves with a radius ratio equal to 1 had a significantly negative correlation with the radius. According to several radius combinations, when the radius ratio was greater than 1, the smaller the radius, the better the improvement effect. Conversely, when the radius ratio was less than 1, the larger the radius ratio, the better the improvement effect. However, regardless of the radius combination changes, the improvement effect of perceived speed deviation on the second curve was weaker than that on the first curve.

Pearson correlation analysis was also conducted, and the results are shown in Table 8.

**Table 8:** Correlation analysis of D of s-shape curves (r).

Point	Radius Ratio	R1	R2
TS	--	0.960(0.01)	--
MC1	--	0.262	--
SS	-0.719(0.05)	-0.721(0.05)	-0.297
MC2	0.395	--	-0.876(0.05)
ST	0.244	--	-0.836(0.05)

The results showed that perceived speed deviation at all points except MC1 was significantly correlated with the curve radius (r>0.700, Sig.<0.05). The influence of the second curve radius on the driver’s speed perception was slightly lower than that of the first curve radius. Fig 10(A) shows that perceived speed deviation of the s-shape curve with a radius of 4200 m changes more gently than other curves. After this curve was removed, the correlation was conducted again, and the perceived speed deviation at MC1 was significantly correlated with R1 (r = -0.812, Sig.<0.05). This may be because when driving on curves with large radius, the curvature changes little in the driver’s field of vision while the driver’s speed also changes little, causing a weak improvement in the perceived speed accuracy. Moreover, it can be further explained that when the radius is large enough (4200m), the perceived speed of the driver will not be affected by the radius.

#### Main influence factors on speed perception

This study classified the freeway curve types, focusing on the influence of radius, the curve combination, and the tangent length between curves on drivers’ speed perception. The specific results were as follows.

(1) Influence of radius on speed perception
  As can be seen from the curve data above, radius is the most important factor affecting the driver’s speed perception in the curve, especially for single and s-shaped curves. There is a significant positive correlation between perceived speed and radius, and a significant negative correlation between perceived speed deviation and radius at the MC and ST points in curves.
(2) Influence of curve combination on speed perception
  The adjacent curve in one direction with 400m tangent length and the reverse curve with 400m tangent length were compared to discuss the influence of curve combination on driver’s speed perception. It was found that the curve combination has no significant influence on the improvement effect of speed perception when the tangent length between curves was the same. The correlation analysis of the perceived speed deviation at each point and the curve combination also found that there was no obvious correlation. This indicates that the curve combinations involved in this study had no effect on drivers’ speed perception.
(3) Influence of tangent length between curves on speed perception
  The s-shape curves were regarded as curves where the tangent length between curves was 0. They were compared with the adjacent curve in one direction and the reverse curves of same radius to further analyze the correlation between perceived speed deviation and tangent length, as shown in Table 9.

**Table 9:** Correlation analysis between tangent length and perceived speed deviation (r).

TS1	MC1	ST1	SS/MT	TS2	MC2	ST2	ST2-TS1
0.125	-0.455	0.053	-0.125	-0.252	-0.502	-0.534(0.05)	-0.506

ST2-TS1: The overall increase in perceived speed deviation in curves.

The results showed that the length of tangent between curves had a greater influence on the driver’s speed perception when driving on the second curve. At MC2 and ST2 points, the perceived speed deviation and tangent length were moderately negatively correlated (r>0.500), and the overall improvement of speed perception accuracy of the curve is moderately correlated with the tangent length (r = -0.506). In other words, the shorter the tangent length was, the better the speed perception accuracy of the overall curve was for drivers. Combined with the driver’s speed change on the compound curve, the analysis showed that the longer the tangent length, the more obvious the driver’s speed recovery, and the greater the speed change. This may also be one reason for drivers’ more accurate speed perception in curves with shorter tangents.

(4) Influence of drivers’ personal attributes on speed perception
  Through the analysis of drivers’ perceived speed deviation and drivers’ personal attributes at each feature point of the single curve, it could be seen that the speed perception of drivers at TD and MC points was significantly correlated with their driving experience. When entering a curve, the more experienced the driver, the more accurate the speed perception deviation.

## Discussion

Based on various types of curves on the freeway, this study systematically analyzes the characteristics of drivers’ speed perception by collecting actual and perceived speed data and evaluates the effectiveness of the driving simulation test method.

Many researchers have evaluated the effectiveness of driving simulators in relevant studies, and they have proved that driving simulators can provide enough visual information for the driver to correctly perceive the speed and distance [24,25]. However, other researchers have pointed out that when drivers drive at a higher speed, their driving speed in the driving simulator increases slightly compared with the actual road speed [31,32]. The difference in driving speed tendency may be due to the difference in speed perception at different speeds, which may affect the absolute effectiveness of the driving simulator. This study combines the simulated driving test and field test, and by comparing the actual speed and perceived speed data, it proves that the driving simulation can fully represent the speed selection characteristics and speed perception characteristics of drivers on freeway curves.

Previous studies have found that when driving at high speeds, drivers generally underestimate the speed [14], which has been confirmed in this study. Similarly, when driving on curves, drivers also underestimated the speed at high speeds. From the drivers’ actual speed data obtained in this study, drivers generally drive at a high speed in curves, and the speed entering the curve exceeds the speed limit regardless of the radius. This may be a factor in accidents caused by drivers speeding on curves, because the faster the speed, the higher the accident rate and the more serious the accident consequence [33]. Moreover, by observing the speed perception deviation value of drivers when they just enter a curve, we found that when the radius was greater than 650 m, the speed perception deviation was lower than that when the radius is less than 650 m, with the actual speed being higher. This result is consistent with Recarte’s finding that the passengers’ estimation deviation of speed decreases with the increase in speed [18].

When analyzing the speed perception characteristics of single curves, we found that the TS point was the position with the largest speed perception deviation and the most serious degree of underestimation. Subsequently, the speed perception deviation of the MC and ST points gradually decreased and even were overestimated when the curve radius was small. There are two explanations for this situation. First, curves provide drivers with more peripheral vision stimulation than straight roads, and at a constant distance. When the distance between stimuli and the observer’s motion velocity is constant, the observer can more easily determine specific speed information [34]. Second, from the perspective of speed change, when drivers just enter a curve, their speed decreases dramatically. Thus, to accurately adapt to the curve and ensure safety, they need to drive at a lower speed. Moreover, when drivers are about to leave the curve, they see that the following road geometry is smoother and are eager to regain lost speed before entering the next curve. According to the theory of "speed adaptability," drivers overestimate the speed after accelerating and underestimate the speed after decelerating [15,16]. From this point of view, the acceleration and deceleration behaviors of drivers in curves also affect the speed perception of drivers to some extent.

By analyzing the driving and perceived speed data of the compound curve, we found that the tangent between the curves plays an important role in speed recovery; the longer the tangent, the more significant the speed recovery. Conversely, in terms of speed perception, the shorter the tangent is, the better the accuracy of the driver’s speed perception. Hence, speed recovery caused by the tangent length may be one of the factors affecting the accuracy of the driver’s speed perception. In contrast to existing literature, we found that the parameters of the tangent length between curves are generally conservative in road design [35].

In Fildes previous research, he found that experienced drivers had better speed perception accuracy. This was also confirmed in this study. More experienced drivers had more accurate speed perception accuracy when entering curves (TS and MC points). But the correlation was not significant when they leaving the curve (ST point).

## Conclusions

The main conclusions of this study are as follows.

First, it is reliable to use the driving simulation method to study drivers’ speed perception in curves. Its absolute and relative validity have been verified. Second, the drivers’ estimation of speed on freeway curves is inaccurate, especially the inexperienced drivers. Along with the common phenomenon of high-speed driving, the speed is also widely underestimated. Third, for single curves, drivers underestimate the speed to the greatest extent at the TS point, then the underestimation degree gradually decreases. Conversely, the speed estimation at the ST point tends to be accurate and is even overestimated when the radius is small. Fourth, there is a superposition effect on the speed perception between two adjacent curves; however, the combination between curves has no significant influence on the speed perception. Fifth, for the s-shaped curve with two curves of the same radius, the smaller the radius, the better the improvement effect of speed perception accuracy. Moreover, the speed perception improvement ability of s-shape curves with a large radius followed by a small radius is better than those with a small radius followed by a large radius. Finally, the tangent length between curves mainly affects the speed perception in the second curve. With the increase in the tangent length, the accuracy of the driver’s speed perception gradually decreases. This may be because longer tangents allow the driver to recover more speed.

In summary, this study evaluated the impact of curve characteristics on the driver’s speed perception and provided reference for reasonable speed control In the formulation of speed limit management measures, not only the geometric and roadside environment should be considered, but also the deviation between the driver’s perception and the actual speed can not be ignored. Especially in the position just entering the curve, the driver’s speed is greatly underestimated, which may adversely affect traffic safety.

Nevertheless, the current study has the following limitations. First, the selected indicators of the research elements (radius, tangent length between curves) are not strong in continuity, and in order to control the influence of the spiral, the index of the spiral was controlled. Second, due to the complexity of the practical operation of human natural driving test, it is not convenient to carry out large sample test considering the safety of drivers. At the same time, it is necessary to ensure the consistency of simulation and field samples. Hence, a small sample was used in this study. Although the sample size is small, age and gender distribution are considered in the selection process, which has certain typicality. Third, considering that speed perception may be related to drivers’ personality characteristics, future research can explore the impact of personality characteristics on drivers’ speed perception.

## Supporting information

S1 Text Drivers’ speed perception data. (DOCX)
