# Supplementary material for: The influence of freeway curve characteristics on drivers’ speed perception accuracy
Source: PLoS One. 2022 May 4;17(5):e0267250. doi: 10.1371/journal.pone.0267250 (PMC9067667; doi:10.1371/journal.pone.0267250)
Supplement: S1 Text — (DOCX) [file pone.0267250.s001.docx]

**S1 Text**. <https://figshare.com/articles/dataset/Speed_Data_xls/17060081>
